# Glycogen Metabolism and Its Role in Growth and Encystation in *Entamoeba histolytica*

**DOI:** 10.3390/life13071529

**Published:** 2023-07-08

**Authors:** Jordan Wesel, Cheryl Ingram-Smith

**Affiliations:** 1 Department of Genetics and Biochemistry, Clemson University, Clemson, SC 29634, USA; jwesel@g.clemson.edu; 2 Eukaryotic Pathogens Innovation Center, Clemson University, Clemson, SC 29634, USA

**Keywords:** glycogen, *Entamoeba*, glycogen synthase, glycogen phosphorylase, encystation

## Abstract

*Entamoeba histolytica* is a parasitic protozoan that causes diarrheal disease in approximately 100 million people worldwide every year. *E. histolytica* has two forms, the growing trophozoite and the infectious cyst. Trophozoites colonizing the large intestine form cysts that are released into the environment. The ingestion of the cysts in contaminated food and water continues the disease cycle. Here, we investigated the role of glycogen in trophozoite growth and encystation. Glycogen is thought to provide precursors for the synthesis of chitin, a major component of the protective cyst wall. We propose that glycogen also serves as an energy source during metabolic adaptation to different nutrient environments. We examined the role of glycogen in *E. histolytica* by analyzing the growth and encystation of RNAi strains with reduced expression of the single gene-encoding glycogen synthase (*GYS*) or two of three genes encoding glycogen phosphorylase (*PYG*). The *GYS* RNAi strain had a greatly reduced glycogen accumulation, and both the *GYS* and *PYG* RNAi strains exhibited reduced growth in the glucose-poor medium. Both RNAi strains also showed reduced cyst production. Our results suggest glycogen synthesis and degradation are vital to the growth and adaptation of *E. histolytica* to a low-glucose environment such as that encountered in the large intestine.

## 1. Introduction

*Entamoeba histolytica* is a food- and water-borne parasite that causes amoebic dysentery in ~100 million people each year [1]. *E. histolytica* infection is common in nations without proper water treatment and waste disposal infrastructure, and the infection rate can be as high as 50% in some areas [2,3,4,5,6]. Although only ~10% of infections are symptomatic [7,8,9], asymptomatic individuals spread disease by shedding millions of parasites a day [10]. Most *E. histolytica* infections are localized to the intestine, but, in some cases, the infection spreads to the liver, leading to an amoebic liver abscess, which kills up to 50–100,000 people yearly if left untreated [1,11].

*E. histolytica* possesses a uniquely limited metabolic program [12,13]. This amitochondriate parasite lacks a functional citric acid cycle and oxidative phosphorylation [12] and relies mainly on glycolysis [14,15]. *E. histolytica* has a non-canonical pyrophosphate-dependent glycolytic pathway that includes a pyrophosphate-dependent phosphofructokinase to conserve ATP [15,16]. Overall, *E. histolytica* generates five ATP per glucose molecule converted to acetate versus three ATP when ethanol is the end product [17].

*E. histolytica* colonizes the large intestine, a low-glucose environment [18,19]. *E. histolytica* can obtain nutrients from the amino acid catabolism and phagocytosis of intestinal bacteria and cells sloughed from the intestinal lining [12,20,21,22] but is thought to depend, in large part, upon stored glucose in the form of glycogen [23,24]. Glycogen is abundant within the cell, accounting for up to 3 M stored glucose. Glucose-6-phosphate (G6P) is broken down via glycolysis for ATP production but can serve as the precursor for glycogen production in times of glucose abundance. G6P is first converted to glucose-1-phosphate (G1P) by phosphoglucomutase and then to UDP-glucose by UDP-glucose pyrophosphorylase. UDP-glucose then serves as the substrate for glycogen synthase to add glucose units to the growing glycogen molecule. When glucose is needed, glycogen phosphorylase and the glycogen debranching enzyme operate in concert to free G1P (or glucose at the branch points), which is converted back to G6P by phosphoglucomutase for glycolysis.

In addition to sustaining *Entamoeba* growth [25], glycogen is also proposed to provide the precursor for the synthesis of chitin [26,27], a major component of the *E. histolytica* cyst wall [28]. Glycogen levels are high in *E. histolytica* trophozoites growing in a glucose-rich medium but are rapidly depleted in a glucose-poor medium, and cysts have little glycogen [26,27]. We have shown that *E. histolytica* encystation is induced by the removal of glucose from the culture medium [29].

To determine the role glycogen plays in the ability of *E. histolytica* to adapt to an environment with limited glucose, we characterized the growth, glycogen levels, and encystation in the wild-type strain and RNAi strains silenced for glycogen synthase, *GYS* (EHI_085980), or for two of the three putative *E. histolytica* glycogen phosphorylase genes, *PYG1* (EHI_138380) and *PYG2* (EHI_096930). We found that the RNAi strains were less able to adapt to nutritional downshifts, and encystation was impaired. Additionally, we found that expression of the genes encoding glycogen synthase and glycogen phosphorylase are coordinately regulated. Our results indicate that the ability to synthesize and break down glycogen plays an important role in *E. histolytica* during growth and encystation.

## 2. Materials and Methods

### 2.1. Materials

The chemicals were purchased from Sigma-Aldrich, VWR International, Fisher Scientific, and Life Technologies. Heat-inactivated adult bovine serum was purchased from Gemini Bio (www.gembio.com). Penicillin–streptomycin solution and Diamond vitamins were purchased from Life Technologies (Carlsbad, CA, USA). Other media components were purchased from Fisher Scientific and VWR.

### 2.2. E. histolytica Culture Maintenance

*E. histolytica* HM-1:IMSS was grown axenically in standard TYI-S-33 medium [30]. This medium is designated as TYI glucose, as it contains 50 mM added glucose. TYI basal medium, lacking added glucose, is designated as TYI basal. The cells were grown at 37 °C in 25 cm^2^ untreated tissue culture flasks containing 52 mL of medium.

### 2.3. Vector Construction and Transfection

Constructs for trigger-mediated RNAi gene silencing of the glycogen synthase and the glycogen phosphorylase genes were created using the pKTM3 vector [31] (kindly provided by Dr. Upinder Singh, Stanford University). The *GYS* coding region (EHI_085980) and ~1000 bp of the coding region of the *PYG1* and *PYG2* genes (EHI_138380 and EHI_096830, respectively) were amplified from *E. histolytica* genomic DNA using the KOD Hot Start DNA polymerase kit (MilliporeSigma; Burlington, MA, USA) with the primer pairs shown in Table 1, and cloned into the pKT3M vector using the NEBuilder HiFi DNA Assembly kit (New England Biolabs; Ipswich, MA, USA).

*E. histolytica* trophozoites were transfected with the RNAi constructs by electroporation, as described by Hamann et al. [32], using 2.4 × 10^6^ cells and 100 µg of plasmid DNA. The RNAi strains were maintained under G418 selection (final concentration of 6 µg/mL). An RNAi strain with pKTM3 containing the *LUC* gene was used as a control [31].

### 2.4. RNA Isolation and RT-PCR

RNA was isolated using Trizol Reagent (Invitrogen; Carlsbad, CA, USA) and DNase treated using the TURBO DNA-free kit (Invitrogen; Carlsbad, CA, USA) to eliminate residual DNA. Gene expression was examined by reverse transcriptase PCR (RT-PCR) using the Quantabio qScript XLT 1-Step RT-PCR Kit (Quantabio; Beverly, MA, USA) with the primer pairs shown in Table 1. The expression of the small subunit ribosomal RNA gene (accession number: X61116) was used as the control. The semi-quantitative RT-PCR gels were analyzed using Fiji [33]. Standard PCR using the control primers was used to confirm the absence of genomic DNA in the RNA preparations.

### 2.5. Growth Curves

The cultures were inoculated with 5 × 10^4^ cells in TYI glucose or TYI basal medium and grown at 37 °C. All the growth curves were performed with three biological replicates. Three replicates were harvested and counted at each time point. Trypan blue exclusion was used to distinguish the viable cells [34]. Significance testing was performed using an unpaired Welch two-sample *t*-test with a 95% confidence interval in R studio, R version 4.0.2 (22 June 2020).

### 2.6. Glycogen Determination

The log-phase trophozoites grown in the TYI glucose medium were transferred into fresh medium for 24 h, harvested, and cultures for glycogen determination were inoculated at 1 × 10^4^ cells/mL. The cells were harvested at the indicated times and frozen at −80 °C until processing. All the time points were performed with the three biological replicates.

The cells were lysed by five cycles of flash freezing in liquid nitrogen, followed by thawing at 37 °C. The lysed suspension was centrifuged at 10,000× *g* for 10 min, and the supernatant was stored at −20 °C.

The cellular glycogen content of the cells was assayed using the Abcam Glycogen Assay Kit II colorimetric assay (www.abcam.com), based on the manufacturer’s instructions, with 30 min hydrolysis and 30 min color development periods. Mock reactions lacking the hydrolysis enzyme were performed to determine cellular glucose, which was background subtracted. Three biological replicates were performed for each strain and each condition tested, and three technical replicates were performed for each assay. Significance testing was performed using an unpaired Welch two-sample *t*-test with a 95% confidence interval in R studio, R version 4.0.2 (22 June 2020).

The total protein concentration in the cell lysates was quantitated by the Bradford method [35] using the Bio-Rad Protein Assay Kit II (Bio-Rad; Hercules, CA, USA).

### 2.7. Encystation

Encystation was performed as previously described [29]. Briefly, trophozoites were grown in 52 mL of TYI glucose medium at 37 °C in 25 cm^2^ untreated tissue culture flasks with plugged seal caps until in mid-log-phase growth (~2 days). The cultures were placed on ice for 10–20 min to release adherent cells and were harvested by centrifugation at 500× *g* for 5 min. The cells were resuspended in a small volume of phosphate-buffered saline (PBS) and counted using a Luna automated cell counter (Logos Biosystems, Annandale, VA, USA). To induce the encystation, the trophozoites were inoculated at 5 × 10^4^ cells/mL into tightly sealed 25 cm^2^ tissue culture flasks (untreated, plugged seal caps) containing 52 mL of TYI basal medium, incubated at 37 °C, and harvested by centrifugation at the indicated times. The cells were analyzed by flow cytometry as described below. The encystation experiments were performed with three biological replicates for each strain at each time point.

The encystation was assessed using flow cytometry, as described previously [29]. Briefly, the cells were washed with PBS, stained with 10 mg/mL of AlexaFluor WGA-488 for 15 min, rinsed with PBS, and fixed in 4% (*v*:*v*) paraformaldehyde in PBS for 15 min. The fixed cells were rinsed and resuspended in PBS and analyzed by flow cytometry using a Cytoflex flow cytometer (Beckman Coulter Life Sciences, Indianapolis, IN, USA), with 100,000 events collected per sample. A 488 nm laser was used for excitation. The flow cytometry data were analyzed using FCS Express 7 Flow.

## 3. Results

### 3.1. Cellular Glycogen Content Is Reduced during Growth in the Absence of Glucose

Cellular glycogen is abundant in glucose-grown *E. histolytica* trophozoites [16]. We determined how cellular glycogen content changes in response to changes in glucose availability. Wild-type cells were cultured in either TYI glucose or TYI basal medium for 24 h, transferred to the opposite medium, and the glycogen content was measured at 8 h and 24 h. As the glycogen assay used also measures free glucose, the level of free cellular glucose was measured and background subtracted from the cellular glycogen level. This correction led to negative values in some cases, indicating that the level of cellular glucose exceeded the level of glycogen.

**Figure 1:**
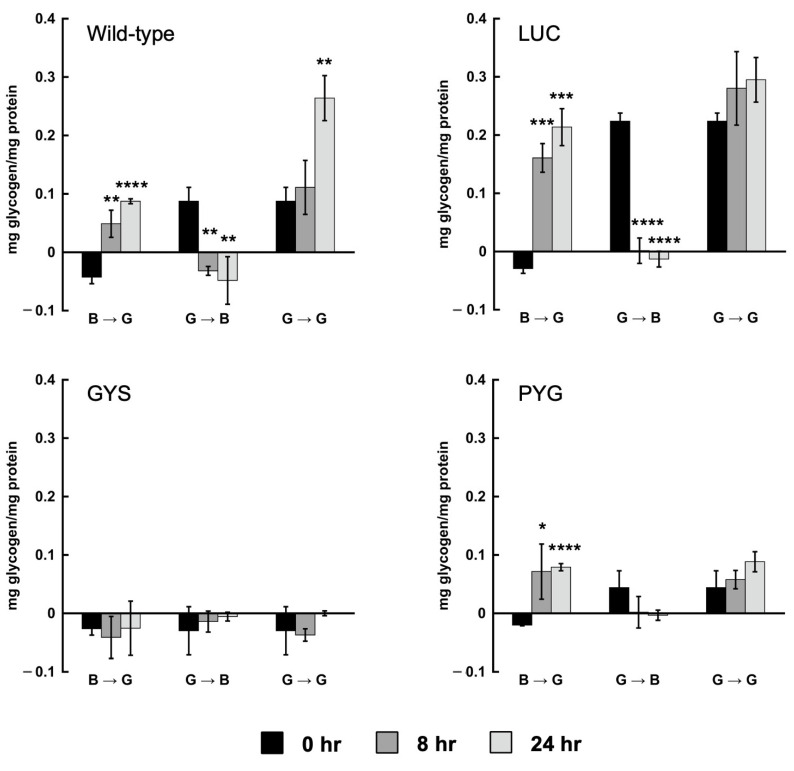
Cellular glycogen content in the wild-type and the *LUC*, *GYS*, and *PYG1+2* RNAi strains during growth under high- or low-glucose conditions. Cells were grown for 24 h in the first medium, transferred to the second medium, and harvested at 0, 8, and 24 h time points. The media transfers were as follows: TYI basal to TYI glucose (B → G), TYI glucose to TYI basal (G → B), and TYI glucose to TYI glucose (G → G). Cellular glycogen content was determined in *E. histolytica* cell extracts by colorimetric assay. Glycogen concentration is normalized to protein content in cell extracts and values are presented as mg glycogen/mg protein. Three biological replicates were performed for each condition and time point, and assays were performed in triplicate. Values are the mean ± SD. Statistical significance was determined for the 8 and 24 h time points relative to the corresponding 0 h time point using an unpaired Welch *t*-test. * *p*-value < 0.05; ** *p*-value < 0.01; *** *p*-value < 0.001; **** *p*-value < 0.0001.

The cells grown in the TYI basal medium for 24 h had no detectable cellular glycogen but accumulated glycogen when transferred to the TYI glucose medium (B → G treatment), with a significant accumulation by 24 h (Figure 1). Conversely, the cells grown in the TYI glucose medium, then transferred to the TYI basal medium (G → B treatment), rapidly used their glycogen stores, such that no detectable glycogen remained after 8 h. In each case, the cells experienced approximately a 3-fold difference in glycogen content between the 0 h time point and the 24 h time point. Surprisingly, the cells maintained in the TYI glucose medium and transferred to fresh TYI glucose medium (G → G treatment) also experienced approximately a 3-fold increase in glycogen by 24 h. The starting glycogen content of these cells was similar to the starting glycogen content for the cells receiving the G → B treatment and the final glycogen content for the cells receiving the B → G treatment. This suggests a continuous supply of excess glucose allows even higher accumulations of glycogen.

### 3.2. Trigger-Mediated RNAi Silencing of the Glycogen Synthase and Glycogen Phosphorylase Genes

*E. histolytica* has one putative glycogen synthase gene (*GYS*; EHI_085980) but has four putative glycogen phosphorylase genes. The deduced amino acid sequences of EHI_138380 (*PYG1*) and EHI_096830 (*PYG2*) are 867 and 884 amino acids, respectively, and share ~66% sequence identity. Two additional putative *PYG* genes, EHI_189300 (*PYG3*) and EHI_043240 (*PYG4*), have deduced amino acid sequences of 467 and 193 residues, respectively. The PYG4 amino acid sequence shares 100% identity with amino acids 621–813 of PYG1. The PYG3 amino acid sequence shares 100% identity with two regions of PYG2, with amino acids 25–236 and 244–467 of PYG3 matching with amino acids 1–212 and 661–884 of PYG2, respectively. Human and mouse glycogen phosphorylases have both a muscle and a liver form. The mouse glycogen phosphorylase amino acid sequences are similar in length and share 45–46% sequence identity with PYG1 and PYG2, and we focused our study on these two putative glycogen phosphorylases.

**Figure 2:**
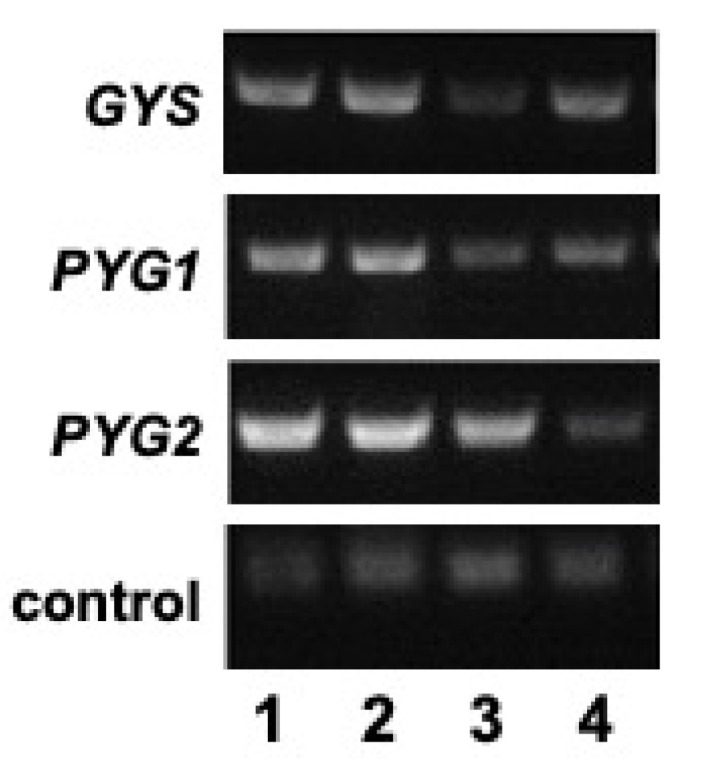
*GYS*, *PYG1*, and *PYG2* expression in RNAi strains. RT-PCR was used to analyze expression of *GYS*, *PYG1*, *PYG2*, and a constitutive control gene encoding a small ribosomal subunit. Wild-type *E. histolytica* (lane 1), *LUC* RNAi control strain (lane 2), *GYS* RNAi strain (lane 3), and *PYG1+2* RNAi strain (lane 4). The RNA was isolated from cells grown in TYI glucose medium for 48 h and treated with DNaseI to remove residual DNA. The results shown are representative of three biological replicates.

To further examine the role of glycogen in *E. histolytica* growth in the absence or presence of glucose, we used trigger-mediated gene silencing to knock down glycogen synthesis or glycogen breakdown. We targeted *GYS* in one RNAi strain and targeted both *PYG1* and *PYG2* in a second RNAi strain (designated as *PYG1+2*). A reverse transcriptase PCR (RT-PCR) analysis indicated partial gene silencing of the targeted genes in their respective RNAi strains versus the wild-type strain, and a control RNAi strain (designated as *LUC*) harboring an RNAi plasmid containing a gene encoding luciferase (Figure 2). Quantitative analysis of the RT-PCR results from three biological replicates for each strain indicated a 60 ± 7% reduction in the *GYS* transcript in the *GYS* RNAi strain and a 43 ± 7% and a 70 ± 9% reduction, respectively, for the *PYG1* and *PYG2* transcripts in the *PYG1+2* RNAi strain versus the *LUC* control strain. Thus, gene silencing was strong but incomplete in the two RNAi strains. The transcript levels of a constitutively expressed control small subunit rRNA gene were found to be similar in all four strains (Figure 2).

### 3.3. Cellular Glycogen Is Reduced in the Glycogen Synthase and Glycogen Phosphorylase RNAi Strains

Since cellular glycogen was quickly depleted in the wild-type strain after transfer to the medium lacking glucose, we examined the cellular glycogen content in the *GYS* and *PYG1+2* RNAi strains and the control *LUC* RNAi strain. The *LUC* RNAi strain behaved similarly to the wild-type strain in both the B → G and G → B treatments, suggesting the presence of the pKT3M plasmid and G418 selection did not cause any substantial change in glycogen production or breakdown. The cells showed a rapid increase in glycogen in just 8 h in the B → G treatment and the depletion of stored glycogen in 8 h in the G → B treatment (Figure 1). The cells maintained a high level of glycogen during the G → G treatment, as expected.

Despite incomplete gene silencing in the *GYS* RNAi strain, the cellular glycogen was below the level of free glucose under all the growth conditions tested, as indicated by the negative values (Figure 1). Surprisingly, the cellular glycogen was reduced in the *PYG1+2* RNAi strain (Figure 1). The cells grown continuously in the presence of high glucose (G → G treatment) maintained a steady level of glycogen, but this level was much lower than that in the control *LUC* RNAi strain. The cells in the B → G treatment did show increased glycogen at the 8 and 24 h time points, but this was reduced compared to the control *LUC* strain. The cells in the G → B treatment depleted their low level of glycogen by 8 h.

### 3.4. Growth of the GYS and PYG1+2 Strains Is Impaired in the Absence of Glucose

**Figure 3:**
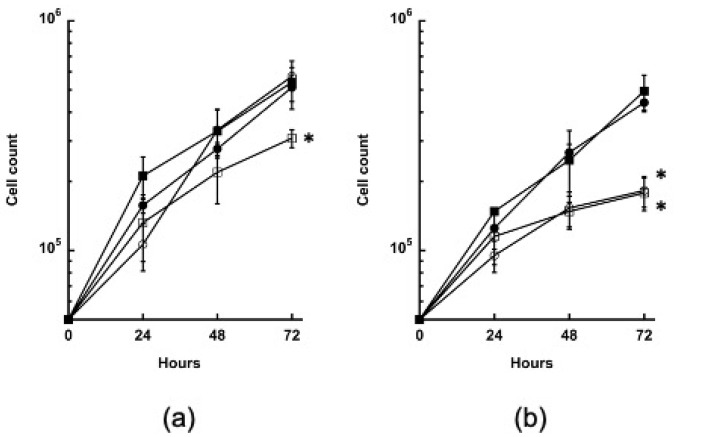
Growth of the *GYS* and *PYG1+2* RNAi strains in a high- versus low-glucose medium. Cultures were grown in (**a**) TYI glucose or (**b**) TYI basal media, and the cells were harvested and counted at 24, 48, and 72 h. Symbols: ● wild-type; ■ *LUC* RNAi; ○ *GYS* RNAi; ☐ *PYG1+2* RNAi. Three biological replicates were performed for each strain, medium, and time point. Statistical significance was determined using an unpaired Welch *t*-test. * *p*-value < 0.05.

The growth of the RNAi strains was examined in the TYI glucose and TYI basal media to determine whether impaired glycogen synthesis or breakdown had any overall effect. The wild-type and the *LUC* and *GYS* RNAi strains grew well in the TYI glucose medium, but the *PYG1+2* RNAi strain had reduced growth (Figure 3a). The growth of the wild-type and the *LUC* RNAi strain were similar in the TYI basal to the TYI glucose medium (Figure 3b), but the *GYS* and *PYG1+2* RNAi strains grew poorly. These results suggest that impaired glycogen synthesis is not deleterious when glucose is readily available, but cells cannot recover well from a nutritional downshift when glycogen synthesis or breakdown is impaired.

### 3.5. Encystation Is Impaired in the GYS and PYG1+2 RNAi Strains

**Figure 4:**
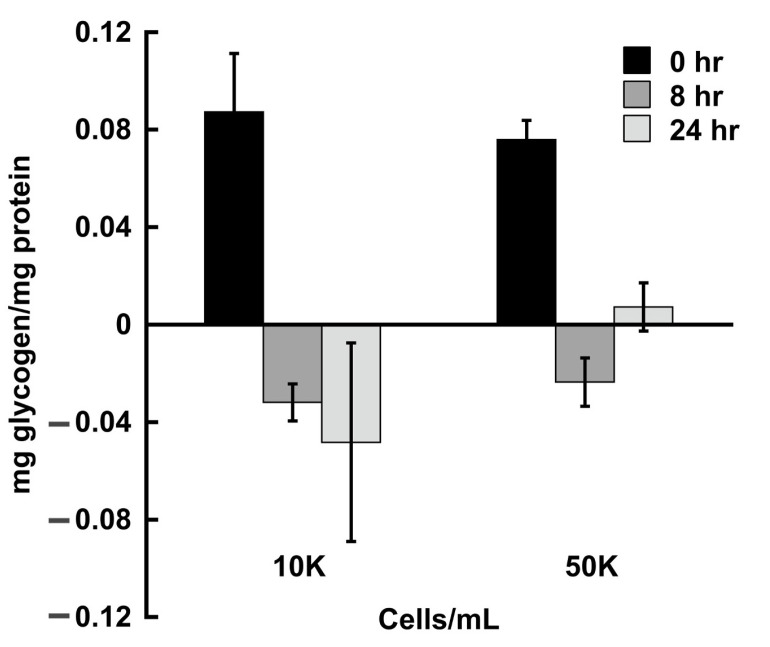
Effect of cell density on glycogen content. Wild-type *E. histolytica* was grown in TYI glucose medium, harvested, and inoculated into TYI basal medium at a starting cell density of 1 × 10^4^ cells/mL (maintenance density, 10 K, **left**) or 5 × 10^4^ cells/mL (encystation density, 50 K, **right**). Cells were harvested at 0, 8, or 24 h, and glycogen content was assayed. Glycogen content in cell extracts is expressed as a ratio of cellular glycogen content to total cell protein content. Assays were performed in triplicate for three biological replicates for each time point. There was no statistical difference between equivalent samples from low versus high cell density.

Glucose deprivation at high cell density induces encystation in the reptile pathogen, *Entamoeba invadens* [36,37]. Cellular glycogen levels have been shown to decrease during encystation, yet glycogen is purported to be the source of glucose for chitin synthesis during encystation [26,27]. We tested whether glycogen is likewise depleted in *E. histolytica* cells induced to encyst. We transferred cells from the TYI glucose medium into the TYI basal medium at an initial inoculum of 1 × 10^4^ cells/mL (used for standard maintenance) or 5 × 10^4^ cells/mL (used for encystation) and measured the glycogen content after 8 h and 24 h. Both the low-density and high-density cultures quickly depleted the stored glycogen when switched to the basal medium (Figure 4) by 8 h, as expected. Surprisingly, the glycogen content increased in the 24 h samples versus the 8 h samples in the high-density culture (*p*-value < 0.05), although overall the glycogen content was still greatly reduced compared to the 0 h sample. The significance of this is unknown.

**Figure 5:**
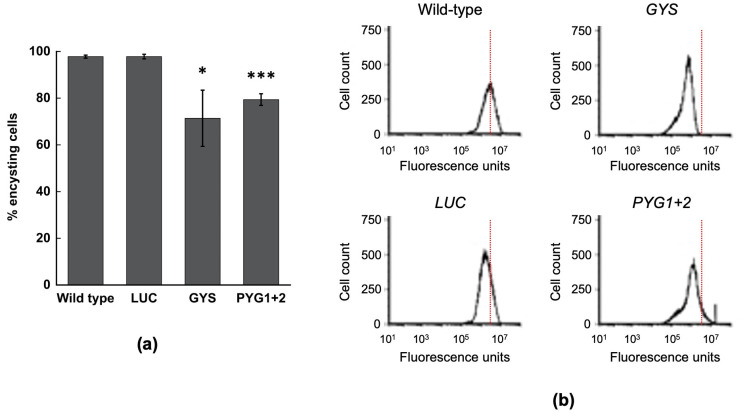
Encystation of the *GYS* and *PYG1+2* RNAi strains. (**a**) The percentage of encysting cells was determined for wild-type *E. histolytica* and the *LUC*, *GYS*, and *PYG1+2* RNAi strains. Strains were cultured at a high cell density (5 × 10^4^ cells/mL) in TYI basal medium (encystation conditions) for 168 h, harvested, stained with AlexaFluor WGA-488, and analyzed by flow cytometry. The results represent three biological replicates for each strain. * *p*-value < 0.05; *** *p*-value < 0.001. (**b**) Representative histograms from 168 h cultures of each strain. The histograms display cell count versus fluorescence intensity; the axes are the same for each histogram. The location of the fluorescence peak for the wild type is marked on each histogram for ease of comparison.

We next examined whether impaired glycogen synthesis and breakdown in the *GYS* and *PYG1+2* RNAi strains affected encystation. The encystation was induced by inoculating log-phase cells grown on glucose into TYI basal medium at a high cell density (5 × 10^4^ cells/mL) [29]. The cells were harvested at 168 h post-inoculation, stained with the chitin-binding lectin, WGA-488, and the progression of encystation based on the change in the cell size and development of a chitin cell wall was assessed by flow cytometry. Our previous work demonstrated that cultures are fully encysted, and the cysts produced are fully mature by 168 h [29]. Cultures of the wild-type and *LUC* RNAi control strains were both nearly fully encysted (97.8 ± 2.2% for each), as compared to the *GYS* and *PYG1+2* RNAi strains, which were 71.4 ± 12.0% and 79.4 ± 2.5% encysted, respectively (Figure 5a). These reductions in encystation for the *GYS* and *PYG1+2* RNAi strains were statistically significant.

The encysted cells were not subjected to sarkosyl treatment, so it is unclear whether the chitin cell wall was fully formed in the encysted cells for the *GYS* and *PYG1+2* RNAi strains. However, a comparison of histograms from the flow cytometry analysis suggests the cysts from these strains may not be mature cysts (Figure 5b). For both the *GYS* and *PYG1+2* RNAi strains, the peak of encysting cells was shifted leftward (lower fluorescence intensity) compared to the wild-type and *LUC* RNAi control strains and displayed a left shoulder. These results may be indicative of cysts that had not reached full maturity, or that lacked an incomplete chitin cell wall. Our results thus indicate that the reduced ability to synthesize or break down glycogen impairs encystation in *E. histolytica*.

### 3.6. Expression of GYS, PYG1, and PYG2 Is Downregulated in the Absence of Glucose

As one final line of inquiry, we examined how *GYS*, *PYG1*, and *PYG2* expression changed when the cells were starved of glucose. An RT-PCR analysis showed that the *GYS* transcript level was reduced in the *PYG1+2* RNAi strain (Figure 2), providing a potential explanation for why cellular glycogen accumulation was reduced in this strain and suggesting that the inability to use glycogen reduces the ability to synthesize glycogen, as well, through some feedback mechanism.

**Figure 6:**
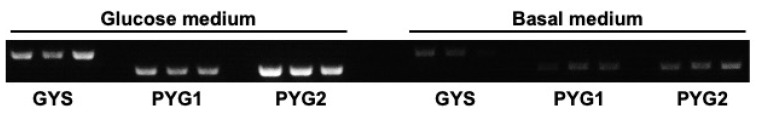
Expression of *GYS*, *PYG1*, and *PYG2* in the wild-type strain grown in TYI glucose or TYI basal medium. Cultures were inoculated with 5 × 10^4^ cells and harvested at 48 h. RNA was isolated and treated with DNaseI to remove residual DNA. RT-PCR was used to analyze expression of *GYS*, *PYG1*, and *PYG2*. The results shown are from three biological replicates for each condition.

We analyzed the transcript levels in the wild-type strain during growth in the TYI glucose or TYI basal medium. When the cells were cultured in the TYI glucose, *GYS*, *PYG1*, and *PYG2* were all expressed, with *PYG2* showing the highest expression (Figure 6). However, when the cells were cultured in the TYI glucose medium and transferred to the TYI basal medium for 48 h, the transcript levels were greatly reduced for all three genes. A semi-quantitative analysis revealed 73 ± 3%, 71 ± 12%, and 78 ± 9% reductions in mRNA levels for the *GYS*, *PYG1*, and *PYG2* genes, respectively, between wild-type cells grown in the TYI glucose medium versus the TYI basal medium. 

## 4. Discussion

Here, we investigated the role of glycogen in *E. histolytica* cell growth and encystation. *E. histolytica* cysts undergo excystation in the small intestine, a nutrient-rich environment. Glucose, the preferred carbon and energy source, is broken down via glycolysis, producing 3–5 ATP per glucose molecule, depending on whether the final product is ethanol or acetate [17]. However, the parasite colonizes the large intestine, a glucose-poor environment [18,19].

Wild-type *E. histolytica* cells grew similarly in the glucose-rich versus glucose-poor medium, indicating they were able to switch to utilizing alternative nutrients for energy when glucose became scarce. Amino acids are amply available in both TYI glucose and TYI basal media and are likely the primary energy source when glucose becomes limited [22]. This metabolic adaptation mimics what is predicted to happen during infection.

We found that glycogen quickly accumulated when *E. histolytica* growing in the glucose-poor medium was transferred to the glucose-rich medium. Conversely, glycogen was rapidly depleted when the cells were transferred from the glucose-rich medium to the glucose-poor medium. *Entamoeba* has previously been shown to store glycogen for use when glucose becomes limited [23,24,25], and our results are consistent with this. Interestingly, wild-type cells grown in the glucose-rich medium and transferred to fresh glucose-rich medium accumulated even higher amounts of glycogen, suggesting that cells will continue to produce and accumulate glycogen to high concentrations during periods of glucose abundance.

*E. histolytica* has one gene encoding a putative glycogen synthase (GYS), the enzyme that catalyzes the addition of glucose units in an α(1 → 4) linkage to a growing glycogen chain. The *GYS* RNAi strain, despite having incomplete gene silencing, had greatly reduced levels of cellular glycogen, even during the growth in the glucose-rich medium. The *GYS* RNAi strain grew well in the glucose-rich medium, indicating that glycogen may be dispensable during growth when glucose is abundant. Unlike the wild-type and the control *LUC* RNAi strains, the *GYS* RNAi strain grew poorly when switched to the glucose-poor medium. One plausible explanation for why this strain was unable to adapt to the glucose-poor environment, presumably by switching to using amino acids as a primary energy source, is because there was insufficient glycogen to serve as an energy source during this period of metabolic adaptation.

*E. histolytica* has four genes encoding putative glycogen phosphorylase enzymes responsible for breaking the α(1 → 4) linkages in glycogen to release glucose 1-phosphate units. Two of these genes, *PYG1* and *PYG2*, encode full-length glycogen phosphorylases, whereas *PYG3* and *PYG4* encode partial sequences of unknown significance. In this study, we focused only on *PYG1* and *PYG2*. An RT-PCR analysis indicated that the silencing of both genes was strong but incomplete. The *PYG1+2* RNAi strain showed reduced growth in both the glucose-rich medium and the glucose-poor medium, unlike the *GYS* RNAi strain. Although this strain did accumulate glycogen when switched from the glucose-poor medium to the glucose-rich medium, the level of glycogen was reduced versus that of the control *LUC* RNAi strain.

The glycogen levels remained low during continuous culturing in the glucose-rich medium, as well, indicating that an impaired ability to break down glycogen also affected glycogen synthesis. The expression of *PYG1* but not that of *PYG2* was found to be strongly reduced in the *GYS* RNAi strain; likewise, the *GYS* expression in the *PYG1+2* RNAi strain also appeared to be reduced. The minimal availability of glycogen in the *PYG1+2* RNAi strain likely coincides with its limited degradation capacity.

A semiquantitative RT-PCR analysis of *GYS*, *PYG1*, and *PYG2* expression in wild-type cells grown in a glucose-rich versus a glucose-poor medium suggests their expression is coordinately regulated. The expression of all three genes was readily observed for the cells grown in the TYI glucose medium, with *PYG2* having the highest expression. In contrast, the expression of all three genes was strongly reduced in the cells grown in the TYI basal medium.

These results suggest that the glycogen synthesis and breakdown processes may be interdependent and commonly regulated, such that the inhibition of one process leads to the inhibition of the other. Glycogen cycling is a major component of *E. histolytica* metabolism, vital to glycolysis regulation. In one study, when glycogen degradation was blocked for two hours, the cellular ATP content dropped to ~50% [15]. As both the *GYS* and *PYG1+2* RNAi strains showed nearly identical patterns of inhibited growth in the absence of glucose, both phenotypes may be due to disruption of the glycogen cycle or to an inability to adapt to the nutrient downshift posed by transfer to a glucose-poor medium. Additional studies are necessary to fully define the interdependence of these processes and coordinate gene regulation.

Due to the proposed dual role of glycogen as both an energy storage molecule and to provide precursors for chitin synthesis during encystation [25,26,27], we investigated whether culturing cells under encystation conditions impacted the cellular glycogen content. Nutrient stress and high cell density are triggers for encystation in *E. invadens* and *E. histolytica* [29,36,37]. The cells inoculated into a glucose-poor medium at low versus high density experienced a similar rapid loss of glycogen by 8 h post-inoculation. The high-density cultures had slightly higher glycogen content at 24 h than the low-density cultures.

One possible explanation for this relates to the fate of the cells. We have shown that cells inoculated at low density can continue to grow in the glucose-poor medium, presumably by switching their metabolism to amino acid breakdown or the use of other nutrients. In this case, glycogen breakdown would supply glucose units in the form of glucose 6-phosphate for glycolysis to provide energy during metabolic adaptation. In contrast, the combination of glucose deprivation and high cell density signals cells to begin encystation. In this case, cells in the high-density cultures may slow down or shut off glycolysis to conserve glucose 6-phosphate as a precursor for chitin synthesis. Thus, although a similar rapid loss of glycogen upon nutritional downshift was observed for both low-and high-density cultures, it may be for very different purposes.

In the “wattle and daub” model of encystation [28], the chitin cell wall of the cyst is made in three phases: (1) the deposition of Jacob lectins on the surface of encysting cells; (2) the chitin synthesis and secretion, followed by the crosslinking of chitin fibrils to Jacob lectins; and (3) the addition of Jessie lectins to solidify and impermeabilize the cyst wall. If glycogen provides the precursor for chitin synthesis, the inhibition of glycogen formation and degradation would be expected to negatively impact the encystation of *E. histolytica* and its ability to form a mature cyst. Indeed, the *GYS* and *PYG1+2* RNAi strains had somewhat reduced encystation overall and may have also produced immature cysts. Although stronger gene silencing in the *GYS* and *PYG1+2* RNAi strains may have a more drastic effect on encystation than observed here, the rapid utilization of stored glycogen upon transfer to a glucose-poor medium argues against glycogen serving as the main source of precursors for chitin synthesis during encystation.

Samanta and Ghosh [27] observed the production of immature, incomplete cysts in *E. invadens* when they silenced the gene-encoding glucosamine-6-P isomerase, reducing chitin synthesis by ~60%. While chitinous cell walls still formed, they appeared incomplete via microscopy, and less bright when examined via flow cytometry. Impaired glycogen synthesis and breakdown likely cause a similar effect. Overall, our results presented here indicate the importance of cellular glycogen in *E. histolytica’s* ability to adapt to a low-glucose environment and form cysts.

## Figures and Tables

**Table 1:** Primers.

	Primer Sequence
Cloning Primers	
GYS RNAi Forward	5′-CGA CTC CCG GGA TGT CAA TTT CTA TCT CAT TAC CTA TTC TAG-3′
GYS RNAi Reverse	5′-CGA CTG GCC CTC GAG TTA AAG TTC AAA AAC AAA GAC TTT TTT C-3′
PYG1 RNAi Forward	5′-ATT GCA GCA TCA ACG CAA GAT GAC CAC ACA AAT TAG ACG-3′
PYG1 RNAi Reverse	5′-TTC ACT TGA TGA GGA TTT TTG CAT GAC TTT TTC ATC TTC TTA AAT CTT CTC ATT ACA-3′
PYG2 RNAi Forward	5′-TGT AAT GAG AAG ATT TAA GAA GAT GAA AAA GTC ATG CAA AAA TCC TCA TCA AGT-3′
PYG2 RNAi Reverse	5′-ACA TTT TAA GTT TAA AAA AGA AGA GTT CAA CAA TCT TCT AAC TAT ATC TTG-3′
RT-PCR Primers	
GYS RT-PCR Forward	5′-TGT CAC CAC AAG AAA TGG CTG-3′
GYS RT-PCR Reverse	5′-AAG GGA AGG AAG TTG TGG CA-3′
PYG1 RT-PCR Forward	5′-GAT GAC CAC ACA AAT TAG ACG ATC AGT TTC TAT G-3′
PYG1 RT-PCR Reverse	5′-GCA AGA GAA TCA AGG AAA CAT GCT GC-3′
PYG2 RT-PCR Forward	5′-GCA AAA ATC CTC ATC AAG TGA AGG AGT TTC TC-3′
PYG2 RT-PCR Reverse	5′-CCA AGT CCT CCT GAT CCA AGT GCA G-3′
ssrRNA Forward	5′-AGG CGC GTA AAT TAC CCA CTT TCG-3′
ssrRNA Reverse	5′-CAC CAG ACT TGC CCT CCA ATT GAT-3′

## Data Availability

All the data relevant to this study are reported within.
